# Pilot Scale Study: First Demonstration of Hydrophobic Membranes for the Removal of Ammonia Molecules from Rendering Condensate Wastewater

**DOI:** 10.3390/ijms21113914

**Published:** 2020-05-30

**Authors:** Brian Brennan, Ciprian Briciu-Burghina, Sean Hickey, Thomas Abadie, Sultan M. al Ma Awali, Yan Delaure, John Durkan, Linda Holland, Brid Quilty, Mohammad Tajparast, Casper Pulit, Lorna Fitzsimons, Kieran Nolan, Fiona Regan, Jenny Lawler

**Affiliations:** 1 DCU Water Institute, School of Chemical Science, Dublin City University, D09V209 Dublin 9, Ireland; brian.brennan27@mail.dcu.ie (B.B.); briciu.ciprian@gmail.com (C.B.-B.); kieran.nolan@dcu.ie (K.N.); fiona.regan@dcu.ie (F.R.); 2 DCU Water Institute, School of Biotechnology, Dublin City University, D09V209 Dublin 9, Ireland; sean.hickey27@mail.dcu.ie (S.H.); linda.holland@dcu.ie (L.H.); brid.quilty@dcu.ie (B.Q.); Mohammad.tajparast@mail.mcgill.ca (M.T.); casper.pulit2@mail.dcu.ie (C.P.); 3 DCU Water Institute, School of Mechanical & Manufacturing Engineering, Dublin City University, D09V209 Dublin 9, Ireland; t.abadie@imperial.ac.uk (T.A.); Sultan-almaawali@hotmail.com (S.M.a.M.A.); yan.delaure@dcu.ie (Y.D.); lorna.fitzsimons@dcu.ie (L.F.); 4 ABP Food Group, E21D588 Kilcommon, Ireland; john.durkan@abpfoodgroup.com; 5 Qatar Energy and Environmental Research Institute, Hamad bin Khalifa University, Doha, P.O. Box 34110, Qatar

**Keywords:** ammonia, hydrophobic, membranes, polypropylene, polytetrafluoroethylene, rendering condensate, wastewater

## Abstract

Hydrophobic membrane contactors represent a promising solution to the problem of recycling ammoniacal nitrogen (N-NH_4_) molecules from waste, water or wastewater resources. The process has been shown to work best with wastewater streams that present high N-NH_4_ concentrations, low buffering capacities and low total suspended solids. The removal of N-NH_4_ from rendering condensate, produced during heat treatment of waste animal tissue, was assessed in this research using a hydrophobic membrane contactor. This study investigates how the molecular composition of rendering condensate wastewater undergo changes in its chemistry in order to achieve suitability to be treated using hydrophobic membranes and form a suitable product. The main objective was to test the ammonia stripping technology using two types of hydrophobic membrane materials, polypropylene (PP) and polytetrafluoroethylene (PTFE) at pilot scale and carry out: (i) Process modification for NH_3_ molecule removal and (ii) product characterization from the process. The results demonstrate that PP membranes are not compatible with the condensate waste as it caused wetting. The PTFE membranes showed potential and had a longer lifetime than the PP membranes and removed up to 64% of NH_3_ molecules from the condensate waste. The product formed contained a 30% concentrated ammonium sulphate salt which has a potential application as a fertilizer. This is the first demonstration of hydrophobic membrane contactors for treatment of condensate wastewater.

## 1. Introduction

Increasing research interest has focused on the use of hydrophobic membrane contactors in recent years as a method of recovering ammonia (NH_3_) molecules from wastewater (WW) streams due to a number of advantages including: fast separation from WW due to the large surface area of the membranes, low energy input per mole of (NH_3_) removed, production of a viable fertilizer as the final product which can be sold, independent control of gas and liquid flow rates, and ease of operation [1,2]. However, to date, no studies have focused on using hydrophobic membrane contactors to treat rendering facility condensate wastewater (RCWW) as the matrix. 

RCWW is a stream of WW rich in (NH_3_) molecules produced from the processing of meat industry waste into viable products which involves the heat processing of animal waste tissue [3]. The purpose of the rendering process is to separate water, fat and protein components and produce stable products of commercial value via steam treatment [4]. RCWW is known to have very high (NH_3_) concentration and has been reported to contain between 500 and 1000 mg/L Total Kjeldahl Nitrogen (TKN) which is the sum of organic nitrogen and NH_3_ molecules [5]. It is crucial that (NH_3_) molecules are treated appropriately before it is released into the environment as even small concentrations such as 0.05 mg/L can result in some fish species suffering from poor growth, fertility issues and making them more susceptible to bacterial infection. Whilst other fish can withstand high concentrations and at 2 mg/L they suffer from tissue damage, extreme lethargy and death [2,6]. The conventional methods to treat (NH_3_) molecules in WW include air stripping, break-point chlorination, selective ion exchange and biological nitrification/denitrification [7]. These methods all have their own benefits but are inefficient as air-stripping is not suitable for low concentrations of (NH_3_) in WW, break-point chlorination requires large treatment volumes, the ion exchange method requires expensive organic resins while the biological nitrification/denitrification method are slow processes that require large treatment vessels as well as significant energy input for air supply to aerobic phases [8]. 

EU regulations require that (NH_3_) levels being released back into the environment must be below 15 mg/L [9,10]. In order to achieve the required legislative limits, an efficient method to remove (NH_3_) molecules must be installed, easily integrated to the WW treatment facility, with low operational and capital costs and short retention time. This makes the use of hydrophobic membrane contactors ideal as various studies have shown as much as 99% (NH_3_) removal from waste streams [11]. The mechanism of separation by hydrophobic membrane contactors is based on the mass transfer between the gas phase and the liquid phase [7]. Ammonium (N-NH_4_) in the feed phase is converted into an (NH_3_) volatile gas phase by increase of pH and/or temperature [1]. The volatile (NH_3_) molecules then diffuse through the membrane pores from the feed side containing the WW to the permeate side containing the stripping solution, which is typically an acid that reacts with the (NH_3_) molecules to form (N-NH_4_) salts (most commonly sulfuric acid (H_2_SO_4_)) [6]. The feed and permeate sides are separated by a hydrophobic membrane sheet which acts as a barrier [12]. Polypropylene (PP) and polytetrafluoroethylene (PTFE) are the most commonly used membranes as they provide good separation to the two phases due to their high hydrophobicity [12]. The driving force of the mass transfer is the difference in NH_3_ concentrations in the 2 phases [13]. The (NH_3_) molecules immediately reacts with the stripping solution on the permeate side of the membrane and forms a non-volatile compound; as such the (NH_3_) molecule levels on the permeate side is essentially zero [7]. 

Membrane wetting has been reported to be a significant issue affecting the feasibility of the use of hydrophobic membrane contactor technology [14]. Certain surfactants in WW such as fat, oils & grease (FOG), dissolved organic matter and colloids lower the liquid surface tension of the feed solution and cause wetting of the membrane pores [15]. Wettability is controlled by the chemical composition of the membrane [2]. The membrane can be characterized under 3 wetting modes; non-wetted, fully wetted and partially wetted. The non-wetted mode is when the membrane pores are completely filled with gas and is the highly preferred mode. Partial wetting is when the liquid can partially enter the membrane pores and causes wetting. Finally, fully wetted membrane pores result in a direct liquid flow from the feed side to the permeate side deteriorating the separation quality [16].

This study is the first demonstration of membrane contactors for the treatment of (NH_3_) molecules from RCWW. The study involves the measurement of the physicochemical characteristics of RCWW and the required pre-treatment steps for the most efficient removal of (NH_3_) molecules from RCWW. The use of PP and PTFE membranes for the removal of (NH_3_) molecules from RCWW, a matrix which has not been examined in the literature to date, was investigated. The membranes were characterized in terms of their surface energy (contact angle (CA), chemical morphologies, surface roughness, porosity and liquid entry pressure (LEP) before and after being exposed to the RCWW at different time intervals. An initial cost analysis of NH_3_ molecule removal using hydrophobic membrane contactors with production of ammonium sulphate ((NH_4_)_2_SO_4_) under different operating conditions is presented. The mass transfer efficiency and wettability of the membranes are determined, and the (NH_4_)_2_SO_4_ product characteristics are discussed.

## 2. Results and Discussion

**Figure 1:**
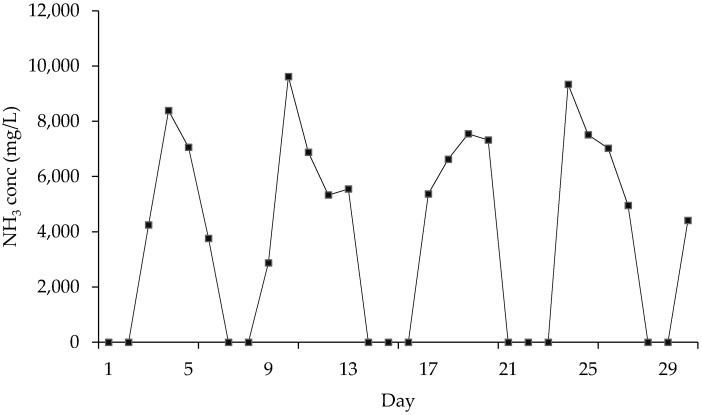
Variation in NH_3_ levels in RCWW over the course of one month (April 2018).

RCWW is a waste product from the processing of animal waste which possess a high (NH_3_) concentration. The volume of animal waste processed daily varies which results in a varied (NH_3_) concentration in the RCWW. Operation of the rendering plant starts on a Monday and it begins to produce RCWW on Monday evening which is then fed into an aeration tank where it is conventionally treated for (NH_3_). From this feed, samples are taken to test on the membrane system. On Fridays, RCWW flow ceases due to the plant being shut down for the weekend. This results in no (NH_3_) being produced over the weekend. Figure 1 shows the concentrations of (NH_3_) over the course of a month which shows the variation which is experienced. It can be seen that the highest levels of N-NH_4_ are experienced mid-week or on Tuesday just after operations have started up again. Levels indicated by zero identify when operations were not running in the plant.

A number of tests were carried out in order to characterise the RCWW according to its physicochemical properties including its pH, temperature and turbidity, and how they exert influence on each other. By doing so, it ensures the RCWW is pre-treated efficiently to be in its volatile form so that it is capable of crossing over the hydrophobic membrane in gaseous form. Investigating the turbidity, it allows for the RCWW to be filtered appropriately in order to prevent it from clogging the membrane pores. The second investigation carried out was completed to determine the suitability of polytetrafluoroethylene (PTFE) and polypropylene (PP) membranes for removing (NH_3_) in RCWW. The rate of (NH_3_) removal was determined by measuring the (NH_3_) levels in the feed inlet and feed outlet (retentate). Failure of membranes was further investigated for membrane wetting using CA and leakage using conductivity analysis. 

### 2.1. Physiochemical Characterisation of RCWW

**Figure 2:**
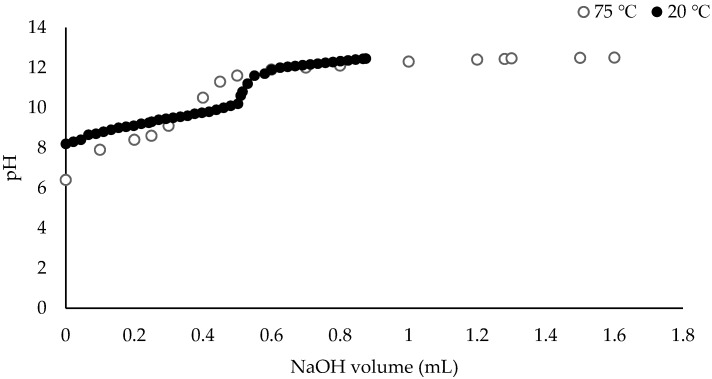
Titration of the same RCWW sample (60 mL) with 30% (*w*/*v*) NaOH at 20 and 75 °C.

An investigation was carried out in order to determine the relationship between the pH and temperature of the RCWW. Two RCWW samples, one with an ambient temperature of 20 °C and the other sample at 75 °C were investigated. As seen below in Figure 2, it was noted that the initial pH drops as the temperature increases. This is caused by equilibrium shifting due to increase ionization of solute molecules according to Le Chatelier’s principle. If the temperature of a dynamic system is increased, the equilibrium will move to lower the temperature by adsorbing the extra heat. That means that the forward reaction will be favoured, and more hydrogen ions and hydroxide ions will be formed increasing the equilibrium constant (Kw). Further tests were carried out with RCWW and deionised water (DI water) to determine the temperature dependence of the pH. Due to this dependence of the pH, an increased volume of sodium hydroxide (NaOH) is required in order to adjust the pH of the RCWW. Figure 2 shows how much NaOH is needed in order to adjust the pH of the RCWW to its optimum conditions (pH of 10.5). This suggests that using a higher temperature (75 °C) will increase the cost efficiency of (NH_3_) removal as less NaOH will be used to increase the pH to 10.5. However, more energy will be used as the RCWW with an initial temperature of 20 °C will need to be heated. However, by increasing the temperature it decreases the potential of membrane clogging which may be more influential in the (NH_3_) removal process as discussed below.

Sample turbidity in RCWW samples was investigated to determine if any pre-treatment is required to protect the membranes from clogging. A decrease in turbidity was noted with an increase in temperature and pH. The RCWW sample heated up to 75 °C and pH adjusted to 12 had a turbidity of 40 NTU whilst the RCWW sample at an ambient temperature and its natural pH (approx. 7–8) had a turbidity of 180 NTU. The results show that there is a strong correlation between an increase in temperature & pH and a lower turbidity and particle size distribution. The RCWW contains protein agglomerates and FOG. Increased temperature and ionization (due to NaOH) has a solubilizing effect on these molecules causing larger particles/agglomerates to break down into smaller constituents which is required to help prevent membrane fouling. It was concluded that operation at high pH and temperatures above 10.5 and 45 °C, respectively, avoid the need for a pre-filter finer than 50 µm to be implemented to protect the membrane contactor, as protein agglomerates and FOG are solubilized under these conditions. As such the optimum pH and temperature of the feed solution should be 10.5 and 45 °C, respectively in order to make the feed solution suitable for the membrane material. In addition, the high pH and temperature reduce the likelihood of biofouling of the membrane.

### 2.2. Effect of RCWW on Membrane Materials

Two commonly used membranes were used in this study, PP and PTFE. The following section discusses the characteristics of these membranes and how they are compromised after being exposed with RCWW. 

#### 2.2.1. Membrane Wetting

Membrane wettability is the ability of a liquid to wet a surface which is controlled by interfacial forces between liquid, solid and vapors [17]. Membrane wetting may be due to inorganic & organic fouling, surfactants, transmembrane pressure or membrane degradation [18]. Three wetting patterns have been identified in the literature in hydrophobic microporous membranes: non-wetting, partial wetting and overall wetting. [16,19]. Compatibility between the membrane type and the liquid absorbent in membrane contactors is an effective parameter in wettability determination. Generally, compatibility depends on absorbent surface tension as well as membrane surface energy [19,20]. Liquids with lower surface tension have greater tendency to wet the surface, while membranes with higher surface energy are more vulnerable to wetting [16]. Organic compounds such as FOG in feed solutions result in a decreased surface tension, and in time, membrane wetting as the hydrophobic organic contaminants begin to adhere to the hydrophobic membrane surface with their hydrophilic component staying in the feed solution which results in a hydrophilic surface on the membrane which results in a decreased CA [18]. CA measurements were used in order to test the hydrophobicity of the membrane surfaces with DI water, RCWW and RCWW with FOG removed.

Table 1 presents a summary of the CA for PP and PTFE membranes with 1 µL of DI water, RCWW and RCWW with FOG removed. The results show that PTFE presents a higher hydrophobicity at 123.1° compared to 115.7° for PP. These results agree with other studies which suggest that PTFE usually poses a rougher surface and a higher CA [21]. The results also show that DI water poses a higher surface tension compared with the RCWW. This decrease in hydrophobicity may be due to a high concentration of organic contaminants present in the RCWW which decreases the surface tension. RCWW which had FOG removed also showed to have a much higher CA which suggests that the FOG present in the RCWW may be causing the membrane surface to become more hydrophilic as suggested by [18]. Figure 1 (supplementary material) represents how the different matrix solutions poses varying surface energy interactions with the membrane surfaces.

**Figure 3:**
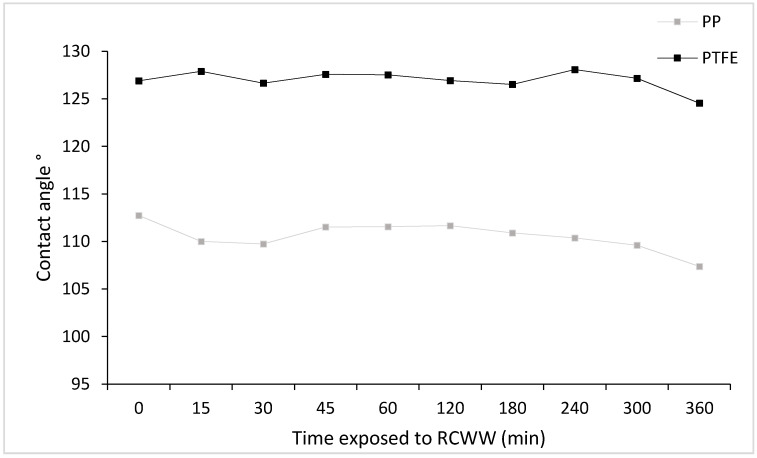
CA for membrane samples (PP & PTFE) exposed to RCWW at different time intervals.

Further investigation was carried out to determine the effect of RCWW to the membrane hydrophobicity over time. PP and PTFE samples were exposed to RCWW at varying times as described in Section 3.2. Initial results (Figure 3) showed that there was no major effect to the membranes hydrophobicity after being exposed to RCWW for at least 6 h as they still remained hydrophobic (above 90°). 

Exceeding the membrane liquid entry pressure (LEP) is another cause of membrane wetting. LEP is a result of the feed solution penetrating the membrane and passing through the membrane to the permeate side. PP and PTFE membranes were investigated to determine their LEP and to investigate how RCWW effected the LEP of the samples over time. Table 2 shows that the LEP of the membranes with no exposure to RCWW is greater than 2 bar. This suggests the membrane materials are suitable for membrane distillation as the LEP of membranes should be at least 1.5 bar in order to be used for MD [22]. Effect of exposure to RCWW suggests that PP membranes are greatly affected by RCWW as their LEP reduces immediately to 0.57 bar and RCWW penetrates the membranes without any pressure thereafter which may be in part due to their large pore size. This suggests that PP membranes are not suitable for MD with RCWW. PTFE membranes performed considerably better as they allowed sufficient LEP values up to 30 min exposure to RCWW. After 30 min the membrane LEP are seen to be below the recommended level and after 120 min they failed without any pressure. However, the membrane system used in this investigation did not exceed 0.6 bar which would allow PTFE to treat RCWW for up to one hour before requiring cleaning. After 60 min of operation the membrane can start to fail due to wetting which may be due to the surface of the membrane being coated; the reduction in surface roughness is evident (Table 3).

#### 2.2.2. Membrane Roughness

**Figure 4:**
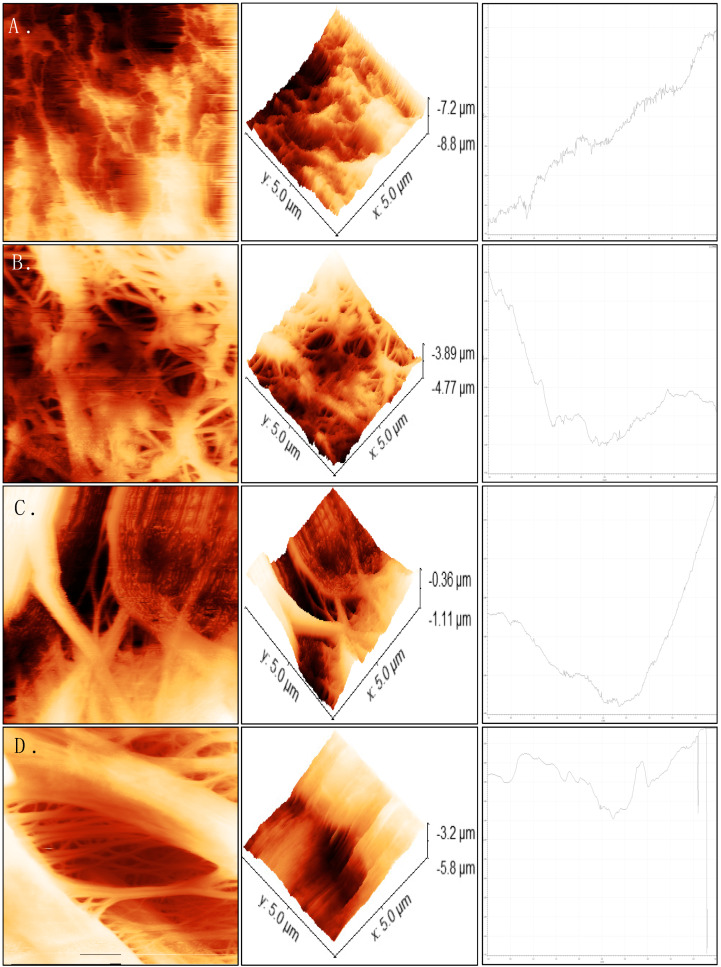
AFM analysis with flat maps, surface height maps and 2D profile for (**A**) PTFE with no RCWW exposure, (**B**) PTFE after 120 min RCWW exposure, (**C**) PP with no RCWW exposure and (**D**) PP after 15 min RCWW exposure.

A quantitative measurement of PP and PTFE was carried out by characterizing the membranes in terms of their roughness (R_a_), root mean square roughness (RMS), mean difference between the highest peak and lowest valley R_max_ and surface area (SA). Atomic force microscopy (AFM) is a powerful tool which allows us to fully understand a samples morphology and 3D images of its topography [21]. In AFM images, the brightest regions represent peaks in the surface and dark regions represent pores or valleys [23]. The images in Figure 4 show (A) PTFE sample with no RCWW exposure, (B) PTFE sample after 120 min RCWW exposure, (C) PP sample with no RCWW exposure and (D) PP sample after 15 min RCWW exposure. 120 and 15 min are presented for PTFE and PP, respectively as they are the points at which membrane failure was first experienced in terms of LEP (Section 2.2.1). Analysis for all other time intervals investigated can be found in the supplementary material (Supplementary Material Figures S1–S4). It should be noted that some time intervals consist of a surface roughness outlier which is assumed to be due to RCWW remaining on the surface on the membrane samples as they are much higher than the initial samples which had no exposure to RCWW (Table 3). The AFM images show variation in surface roughness to samples exposed to RCWW for different time intervals as seen in Figure 4. Both PTFE and PP membrane samples had a root mean square (RMS) roughness value of 249.23 and 154.64 nm, respectively. These results suggest that PTFE have a rougher surface which is in agreement with the values obtained for CA (Section 2.2.1). Other studies have also shown that PTFE typically has a greater surface roughness [21]. Additionally, a study by [24] shows that there is a direct relationship between membrane surface roughness and permeate flux which is in agreement with the PTFE membrane presented in this paper Section 2.3.1. The same author also showed that a membrane with greater surface roughness resulted in decreased membrane fouling. Samples which were exposed to RCWW were shown to lose surface roughness with prolonged exposure (Table 3). The interaction between the particles present in the RCWW and the composite membrane surface caused the roughness to decline. In Figure 4A, samples with no RCWW exposure can be seen to have high peaks and after 120 min exposure (Figure 4B), these peaks are seen to break up and a lower, smoother surface starting to develop. Similar results can be seen for the PP membrane surface. PTFE membranes can be seen to withstand their surface roughness for a much longer period than PP as after 120 min they both seem to have consistent roughness. Overall, the AFM analysis showed that PTFE membrane had a rougher surface than PP, which contributed to the increased flux and longer lifetime. It was also shown that RCWW has a great effect on the membranes surface topography and results in the roughness decreasing over time. Various studies by Vecino et al. [25] showed that oil has a major impact on the surface roughness due to the hydrophobic part of the oil particle adhering to the membrane surface and leaving the hydrophilic part exposed to the sample, which allows the water to enter the membrane pores. Due to the high oily content of RCWW, this may be a major contribution to the surface wetting and thus, appropriate pre-treatments are required in order to remove the oil content from RCWW which was demonstrated by Zheng et al. [26] using omni phobic membranes which showed removal of oil from the feed solution using similar direct contact membrane setup.

#### 2.2.3. Membrane Morphology

**Figure 5:**
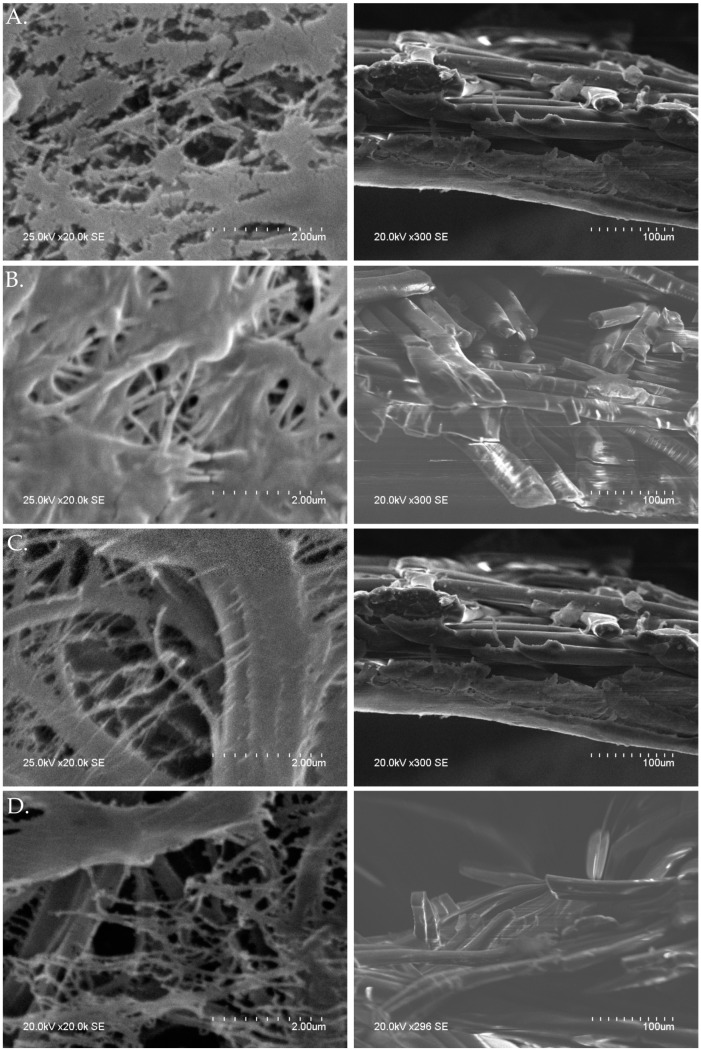
Surface and cross section SEM images for the morphologies of (**A**) PTFE with no RCWW exposure, (**B**) PTFE after 120 min RCWW exposure, (**C**) PP with no RCWW exposure and (**D**) PP after 15 min RCWW exposure.

SEM images shown in Figure 5 present a flat and cross-sectional view of both PTFE and PP before and after RCWW exposure. Similarly to the results presented for AFM analysis, the images for PTFE at 120 min and PP at 15 min RCWW exposure are presented as they represent the maximum exertion according to the LEP results (Section 2.2.1) with the parameters used in the membrane system. The PTFE image with no RCWW exposure (Figure 5A) shows cell like structures with branching and sufficient even pores. The cross-sectional view shows continuous cross-linked fibres. The average pore size for PTFE with no RCWW exposure is 0.39 ± 0.1 µm (Table 4). PTFE samples after 120 min exposure time (Figure 5B) are shown to have a coating on the top as the surface structure is not as defined. Pore size in these samples have decreased (0.3 ± 0.07 µm) which is likely due to pore clogging from the RCWW. The cross-sectional view also shows that fibres are beginning to break which is most likely due to organic matter and particles entering the pores, penetrating the membrane and causing membrane wetting. PP membranes (Figure 5C,D) have a branch like structure with nanoparticles webbed to the larger fibre. Pore sizes (Table 4) appear to remain the same size with and without RCWW exposure. Fibre size can be seen to decrease after exposure and more abundance of nanoparticles webbed are present. The cross-sectional views also show fibres beginning to breakdown. 

Membranes provide a barrier between two liquids and in order to achieve high vapor permeability, the membrane should poses high porosity [27]. Additionally to achieving high flux, a high porosity can also help to prevent membrane wetting [16]. The results for the porosity test are presented in Table 4 and they suggest that the PP membrane is more porous at 86% compared to PTFE at 50%. These results are in agreement with other studies by Shirazi [21]. Statistical analysis using ANOVA also suggested that exposure to RCWW does not affect the membrane porosity as the p-value for PP at 0 and 60 min is 0.27 and PTFE at 0 and 60 min is 0.44 which is greater than 0.05 which suggests there is no significant difference and that exposure to rendering RCWW has no impact on the porosity.

### 2.3. Membrane Performance

#### 2.3.1. (NH_3_) Removal

**Figure 6:**
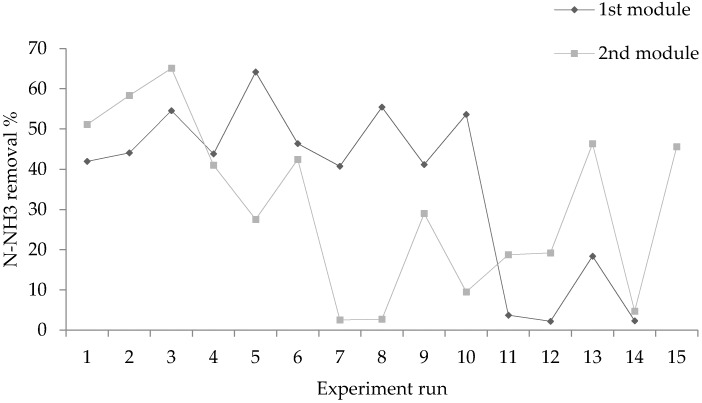
Performance of 1st and 2nd PTFE generation module (Flow feed side 0.05–1 L/h, permeate flow rate 0.05–0.5 L/h, pressure 0.5 Bar and temperature 50 °C).

A series of tests were carried out on the PTFE membrane to investigate the efficiency of (NH_3_) removal from RCWW. RCWW was introduced into the system for testing and reproducibility was achieved by testing 2 modules of the same structure. As seen in Figure 1 the composition of the RCWW is different every day with different concentrations of different components (including (NH_3_) and any possible wetting agents present. As seen in Figure 6, up to 64% (NH_3_) was removed from the RCWW by the 1st module and up to 65% removal was achieved using the 2nd module. These levels of removal show great potential for the use of membrane systems for RCWW treatment as they can be used to reduce (NH_3_) levels and reduce them further by installing a series of membranes in parallel. Additionally this process has a shorter retention time when compared with conventional methods and it produces a viable fertilizer product which can be sold. Module 1 shows to treat the RCWW efficiently for 10 days without cleaning the membrane and after the 10 days its removal efficiency shows to decrease. Module 2 was seen to be efficient for 6 days before its (NH_3_) removal rate started to decrease. The differences in the efficiency of the 2 modules may have been due to the composition of the RCWW sample and an increase in wetting agents present for the 2nd module. However, the membranes present a promising method for treating (NH_3_) in RCWW and could be further optimised by implementing a cleaning procedure for the membranes to prevent them being affected by wetting agents. The occurrence of wetting on the membrane materials can be combatted by introducing a cleaning procedure as described by Chen et al. [28] who used hexane and water in the cleaning in place (CIP) operation in order to clean emulsified membranes at risk of fouling and wetting and showed the flux to return to normal.

The 2nd generation module used in this study was made from PP which failed initial clean water tests due to RCWW being present in the pipework of the system from existing tests. This suggested that PP was not compatible with RCWW and instigated further tests to be carried out to confirm this hypothesis.

#### 2.3.2. Membrane Leakage

**Figure 7:**
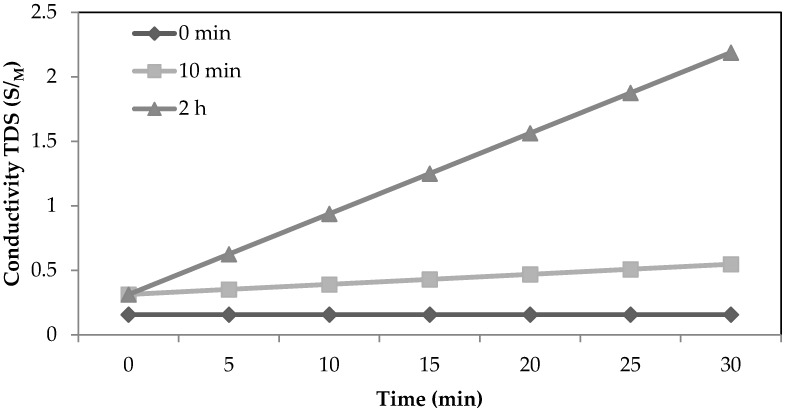
Rate of conductivity increase in the CIP tank after different RCWW exposure times using the PP membrane.

Another, much quicker test to determine membrane integrity is the leaking test. This is carried out by flushing clean water on the feed side of the membrane module in a recirculation mode and sulfuric acid on the permeate side. Conductivity is measured in the feed tank over time. Considering a high conductivity gradient is present between the feed and permeate side of the membrane, if the membrane is leaking, then mass transfer is reversed and an increase in conductivity should be noticed on the feed side and ultimately in the CIP tank. The membrane material was exposed to RCWW at different time intervals (0 min, 10 min and 2 h) and it then underwent operation with clean water and sulfuric acid mixture. If leakage occurred after RCWW it would result in ions from the sulfuric acid passing to the feed side by electrolyte diffusion and increasing the conductivity of the CIP tank. The results after different RCWW exposure times are presented in Figure 7. The results suggest that the much higher conductivity rate after 2 h of exposure as oppose to 10 min exposure which suggests that 2 h exposure is wetting the membrane surface which in turn causes a mixing of the two liquids. 

Results collected from the wetting and leakage tests for PP membranes suggest that PP is not as efficient as PTFE membranes for application to RCWW over long periods of time due to surface wetting. Differences observed between the surface wettability of DI water and RCWW determined that although such PP membrane could be used with other WW types they are not suitable for RCWW without frequent cleaning. In order to implement PP membranes, an appropriate automatic cleaning procedure could be implemented at set times in order to recover the membranes and allow them to be used for longer periods.

### 2.4. Product Characterisation

As stated above, after membrane diffusion NH_3_ reacts with H_2_SO_4_ to form (NH_4_)_2_SO_4_ as seen in Equation (1) [29]. The primary function of (NH_4_)_2_SO_4_ is an agricultural fertilizer in alkaline soils to help promote growth. The ammonium ion is released and it undergoes deprotonation which produces NH_3_ and results in lowering the pH of the soil. It also contributes nitrogen which is essential for plant growth. The sulfur promotes the metabolism of nitrogen, chlorophyll formation and forms amino acids which is the building blocks for proteins [2]. The product formed in this study was found to have a pH of 2–2.5 and a purity of 30%. Liquid products on the market were found to have a purity of 40% making our product a viable and green product, with economic benefit of valorisation of waste produced from the rendering plant [30]. The liquid (NH_4_)_2_SO_4_ may be introduced to the soil by injecting it into the soil has been shown to be carried out by manual injection fertilizing (CULTAN) or by contactless high pressure jet injection [31]. Both methods work perfectly in normal conditions but the contactless method does not reach large depth in very dry soil.

The (NH_4_)_2_SO_4_ solution produced here was found to be very acidic with a pH of 2–2.5. For a product to be of value for application to soils, the pH needs to be increased. A series of titrations were carried out to determine which reagent most efficiently increased the pH of the product (40 mL) based on the costs and quality. The reagents which were used include NaOH, sodium bicarbonate (NaHCO_3_), calcium carbonate (CaCO_3_), RCWW and sludge waste from the aeration tank. Table 5 shows how much of each reagent was required in order to increase the pH from ~ 2 to 7. It was found that RCWW had no effect on changing the pH. The aeration tank sample only changed the pH in one test. NaOH proved to change the pH using the least volume and it also had the smallest standard deviation. NaHCO_3_ and CaCO_3_ required larger volumes and thus NaOH was chosen as the most efficient reagent to adjust the pH of the product in order for it to be applied as land fertilizer.

### 2.5. Molecule and Ion Movement through Membrane Material

**Figure 8:**
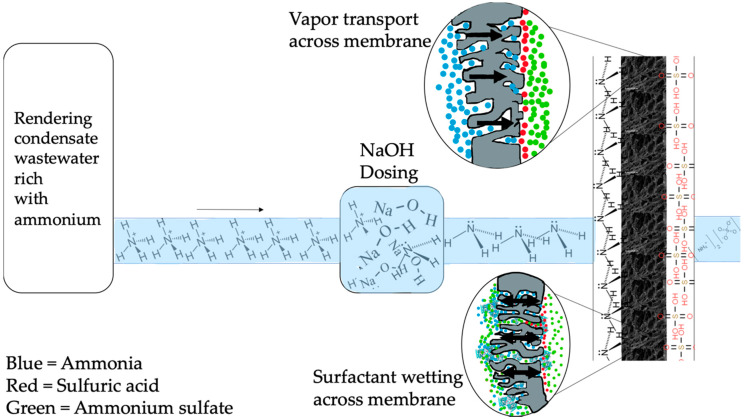
Molecular composition throughout membrane process.

RCWW contains high levels of NH_4_ molecules which must be converted to NH_3_ molecules in order to pass through the hydrophobic membrane (as shown in Figure 8). Once the RCWW solution containing NH_4_ molecules is converted to NH_3_ gas molecules, the NH_3_ molecules are soluble within the RCWW solution and as it approaches the membrane surface it enters the membrane pores due to partial pressure between the feed and permeate side of the membranes (depicted as blue in Figure 8). The NH_3_ vapor molecule passes through the membrane pores and approaches the surface of the permeate side immediately reacting with H_2_SO_4_ molecules (represented by red in Figure 8). (NH_4_)_2_SO_4_ (represented by green in Figure 8) is produced from the reaction at the surface of membrane outside the pores which then circulates back to the permeate tank. As the reaction between these two happens immediately, it results in minimal NH_3_ being present in the permeate side allowing for a high pressure difference between the feed and permeate side which would allow for the diffusion of NH_3_ molecules across the membrane as long as there is ions^+^ for the NH_3_ molecules to react with. Surfactants such as fats and proteins result in wetting of the membrane which causes liquids to penetrate the membrane at the surface and eventually liquid to cross the barrier. Figure 8 shows how the surfactants can cause the membrane to leak and RCWW passes from the feed side to the permeate side and also the H_2_SO_4_ from the permeate to the feed side which results in the reaction to (NH_4_)_2_SO_4_ in both the feed side and across the membrane material.

### 2.6. Preliminary Life Cycle Assessment

A preliminary cost analysis on the membrane system to remove (NH_3_) was carried out and compared with the current method being used to treat RCWW. The operational costs (including chemicals, energy consumption from heaters & pumps and filters) were calculated based on the assumption that the (NH_3_) membrane system is in operation 7 days a week. The costs for the chemicals were based on the volume used in experiments throughout the project and the energy was estimated by measuring the energy usage with an energy meter. The results in Table 6*,* show that the capital cost of the current method is €500,000 and the operation costs (energy for aeration) equate to €1.71 per Kg of NH_3_ removed. No viable product is produced from the current treatment, so no potential revenue is generated on it. The capital cost of the (NH_3_) membrane removal system is €360,000 for the pilot and supply of membranes needed. The operational costs to remove one kg of NH_3_ using the stripping pilot is a total of €2.48. However, the (NH_3_) stripping unit produces (NH_4_)_2_SO_4_ which can then be sold as a fertilizer at a price of €1.54 for (NH_4_)_2_SO_4_ (30%) (*w*/*w*). This would suggest that the cost of treatment by (NH_3_) stripping unit is €0.94 which is cheaper than the current treatment method. However, it should be noted that the current method is a continuous system which has less costs associated with ceasing and starting operation while the tested membrane system was only a batch system. Due to the two systems being different (continues vs batch) it is difficult to make a conclusive comparison. Considering the capital costs and operation costs of the two treatment methods, it can be suggested that preliminary results suggest that (NH_3_) removal using membrane technology is a cheaper method as both capital and operation costs are cheaper.

## 3. Materials and Methods

### 3.1. Materials

**Figure 9:**
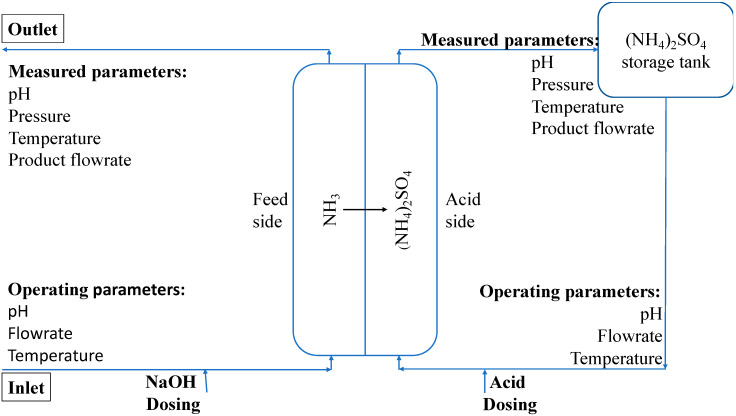
Simplified schematic of process overview (not to scale).

The pilot study was carried out on site in a meat processing plant located in ABP Food Group, Kilcommon, Co. Tipperary, Ireland. Figure 9 shows the conventional method of treatment which is being used in the plant using biological nitrification in an aerobic tank. (NH_3_) rich WW was obtained from RCWW produced by a rendering plant processing animal products. The (NH_3_) stripping unit and membrane materials (PP and PTFE) were designed and sourced from BLUE-tec bv, Industrieweg 16, 6871 KA, Renkum, The Netherlands. Bulk NaOH (30%) (*w*/*w*) and H_2_SO_4_ (96%) (*w*/*w*) which were of analytical grade were procured from Brenntag Chemicals Distribution LTD, Ireland. (NH_4_)_2_SO_4_ (99%) (*w*/*w*%) was procured from Fischer, Ireland and is of an analytical grade.

### 3.2. Characterisation of Membranes

Membrane samples were initially investigated in triplicate prior to being exposed to RCWW and investigated again in triplicate after being exposed to RCWW at different time intervals (0, 15, 30, 45, 60, 120, 180, 240, 300 and 360 min). After exposure to RCWW, samples were sprayed with DI water for 15 s and dried overnight at 103 °C. This investigation allows for the effect of RCWW exposure over time on the membranes to be investigated. The surface and cross-sectional morphologies including the fibre size of both PP and PTFE membranes were obtained using SEM (Hitachi S3400n SEM, Tungsten system). Samples were gold coated and carried out with 20 mV acceleration. The sample pore size and pore size distribution were also investigated using the same SEM instrument and conditions using Image J image analysis software. Surface roughness was quantitatively determined using AFM (BRUKER ICON DIMENSION AFM) using Silicon AFM probes in non-contact/tapping mode (13 kHz resonant frequency and 0.2 N/m force constant), procured from Nano and More, UK. Membrane porosity was performed as outlined by Woo et al., 2017. The surface energy of the PP and PTFE membrane were determined by measuring the CA (FTA200 Dynamic Contact Angle Analyzer) using deionized (DI) water (Elga Purelab Ultra system) in order to determine the hydrophobicity of the membrane surface. LEP is the minimum pressure applied to a dry membrane which results in liquid penetrating inside the membrane pores. LEP was investigated using a LEP set-up with Amicon Test Cells and carried out as outlined by Smolders and Franken [32].

### 3.3. Pilot Operation

The (NH_3_) stripping unit was designed according to requirements and to feature automated, unattended operation, data logging, and remote access. The specifications for two modules evaluated in the investigation are summarized in Table 7. The membrane materials which were used were PP and PTFE.

**Figure 10:**
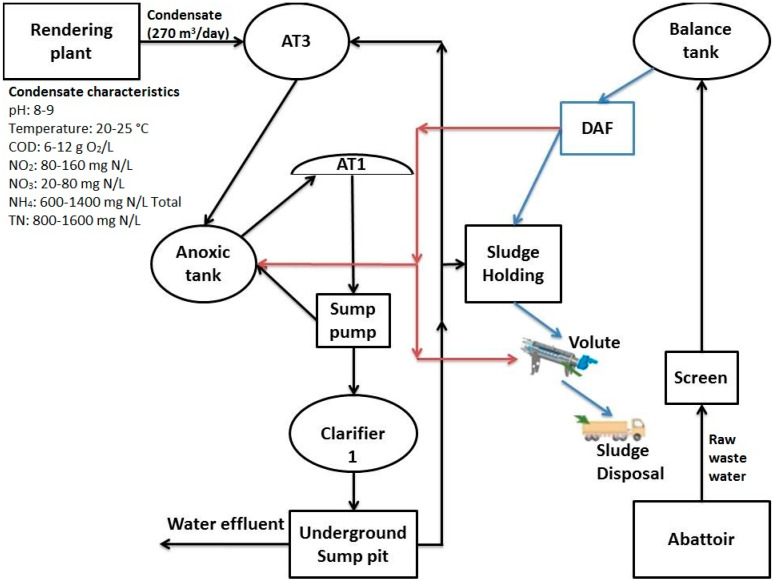
Graphical representation showing the configuration of the current, activated sludge WWTP and characterization of RCWW. AT1 = Aeration Tank 1, AT3 = Aeration Tank 3, DAF = Dissolved Air Flotation.

All experiments were conducted in a pilot scale system module as described briefly in the schematic diagram in Figure 9. The (NH_3_) rich RCWW sample was obtained from the rendering plant waste which is being fed into the nitrification tank (AT3 tank in Figure 10) in the ABP WW treatment facility. The RCWW samples pass through a series of heaters and NaOH dosing pumps to ensure that the temperature and pH are at the optimum conditions to ensure the (NH_3_) is in its volatile gaseous form. The RCWW sample then passes through a settler tank and 50 µL filters to ensure the RCWW sample does not have particles present which may clog the membrane pores. Once the sample is in its volatile gaseous form the RCWW stream is pumped into the feed side of the hollow fibre membrane whilst the stripping solution, H_2_SO_4_, flows along the permeate side of the hydrophobic membrane. The gaseous (NH_3_) diffuses through the hydrophobic membrane and reacts with the H_2_SO_4_ on the permeate side. The reaction that occurs between (NH_3_) and H_2_SO_4_ can be seen in Equation (1) which produces (NH_4_)_2_SO_4_. Both solutions on either side are then recycled to their respective reservoirs which can be seen in Figure 9.

(1) $$2NH_{3}+H_{2}SO_{4}\to {\left(NH_{4}\right)}_{2}SO_{4}$$

### 3.4. Physicochemical Characterisation of RCWW

The RCWW samples were analyzed to characterize them according to their physiochemical properties including pH, temperature and turbidity. By investigating these properties, pre-treatment steps of the RCWW samples were then formulated depending on the results. The temperature was measured using a YSI Proplus^®^ handheld multi-parameter instrument (YSI, Xylem, Hertfordshire, UK) and the pH was tested using a WTW Multi 320 multimeter, pH electrode SenTix 4l. The turbidity of the sample was analyzed using a portable turbidity meter Turb^®^ 430 IR (VWR, Dublin, Ireland). Particle size distribution was carried out using a Malvern Mastersizer 3000E using a Hydro EV wet dispersion unit procured from Malvern Panalytical with a stir speed of 8001200 RPM and sonification of 50%. An investigation was carried out in order to determine the relationship between the temperature and pH of the RCWW samples. Titration experiments were carried out at different temperatures to determine the required volumes of NaOH to raise the pH to optimum levels for (NH_3_) removal. Particle size distribution and turbidity in the RCWW samples was investigated to determine if any pre-treatment is required to protect the membranes from clogging.

## 4. Conclusions

This paper describes the first pilot application of hydrophobic membranes for the removal of (NH_3_) from raw condensate wastewater from the rendering operation of a meat processing plant. Hydrophobic membrane contactors are a relatively new process which have the potential to remove (NH_3_) from WW. RCWW waste is produced from the processing of meat industry waste and is highly concentrated with (NH_3_). To the authors knowledge no literature or studies have focused on the use of hydrophobic membranes for treating (NH_3_) in RCWW waste. In this study, the physicochemical properties of RCWW are characterized, and the optimal pre-treatment steps are determined. The efficiency of PTFE and PP membranes was investigated for NH_3_ removal. The PTFE and PP membranes were characterized and the effect RCWW posed on the membranes was investigated. Additionally, the (NH_4_)_2_SO_4_ product was characterized and the cost comparison between the membrane method and the conventional method was analyzed. The results showed that the pH changes depending on the temperature of the RCWW and as the temperature increases, the pH increases which results in an decreased volume of NaOH being required to achieve the optimum pH to change the NH_4_ into gaseous NH_3_. Characterization of the PTFE and PP membranes showed that PTFE membranes were slightly more hydrophobic with a higher CA, surface roughness and LEP. Characterization studies also showed that membrane exposure to RCWW over time affects the structure of the membranes. Analysis of PTFE membranes showed that the membrane method was efficient and that there was up to 65% removal of NH_3_ removal from the RCWW. However, the PP membrane failed initial water tests and it was concluded that the RCWW contaminated the PP membrane and caused wetting. PTFE membranes may be suitable if they were set up in parallel to allow for multiple treatment steps. The PTFE membranes could also be used as a pre-treatment step to the conventional aeration method to help reduce costs. Additionally, a cost benefit analysis was carried out and showed that the cost of running the NH_3_ stripping unit was more expensive than current treatment methods at €1.71 and €2.48, respectively. However, the NH_3_ stripping unit produces (NH_4_)_2_SO_4_ which can be sold as a fertilizer and reduces the operational product capitalization. Future work should focus on the composition of the RCWW and identify reasons for wetting and fouling of the membrane. Compatible materials which are capable of withstanding the condensate WW should also be identified for hydrophobic membrane fabrication.

## Figures and Tables

**Table 1:** CA values obtained for PP and PTFE membranes using different matrixes.

Matrix	PP	PTFE
DI water	115.7° ± 2.3°	123.1° ± 2.4°
RCWW	92.2° ± 3.4°	99.4° ± 3.1°
RCWW with no FOG	107.3° ± 1.3°	111° ± 2.6°

**Table 2:** LEP values obtained for PP and PTFE samples exposed to RCWW at different time intervals.

Time (min)	0	15	30	45	60	120
PP (Bar)	> 2	0.57 ± 0.12	-	-	-	-
PTFE (Bar)	> 2	> 2	1.67 ± 0.06	1.07 ± 0.06	0.7 ± 0.1	0.07 ± 0.01

**Table 3:** Quantitative analysis of PP and PTFE membrane surface roughness.

Time (min)	PP RMS Roughness (nm)	PTFE RMS Roughness (nm)
0	154.64	249.23
15	134.48	164.4
30	132.86	-
45	511.33 *	131.61
60	118.92	120.42
120	962.2 *	114.87
180	113.66	109.74
240	255.98 *	94.98
300	109.49	556.12 *
360	86.71	85.57

* RCWW residue may have remained on the membrane surface and caused high surface roughness value.

**Table 4:** PTFE and PP membrane morphology characteristics and porosity.

Time (min)	PTFE			PP		
	Porosity (%)	Pore Size (µm)	Fibre Diameter (µm)	Porosity (%)	Pore Size (µm)	Fibre Diameter (µm)
0	50 ± 1	0.39 ± 0.1	23.2 ± 2.9	86 ± 3	0.43 ± 0.1	12.1 ± 1.8
15	-	-	-	-	0.41 ± 0.17	9.1 ± 1.4
60	49 ± 1	-	-	83 ± 1	-	-
120	-	0.3 ± 0.07	17 ± 3.6	-	-	-

**Table 5:** Titrations to investigate most efficient reagent to increase pH of product (titrand = 40 mL).

Reagent	Run 1 (mL)	Run 2 (mL)	Run 3 (mL)	Average (mL)	St. Dev (mL)
111 g/L NaOH	280.00	220.00	200.00	233.33	41.63
15 g/L NaHCO_3_	3520.00	6300.00	4860.00	4893.33	1390.30
9.6 g/L CaCO_3_	690.00	865.00	No change	777.50	123.74
RCWW	No change	No change	No change	-	-
Sludge	540.00	No change	No change	540.00	0.00

**Table 6:** Summary of cost benefit analysis (^a^ 0.009 € kWh^−1^ – was used to estimate the cost. The energy usage (€/kg N) was calculated from the total ammonia removed/year and total blower energy use/year (data obtained from 2016 plant site)).

	(NH_3_) Removal Using Membrane Technology (70% Efficiency)			Current Treatment	
CAPEX	€300,000 (full scale) + €36,000 (membranes)			€500,000 (Aeration tank + diffusers, blowers)	
OPEX	(€/kg N)			(€/kg N)	
	NaOH	H_2_SO_4_	Energy ^a^	Energy	Bio- augmentation
	1.54	0.71	0.26	1.065	0.645
	Maintenance costs (pre-filters)			Maintenance cost	
	0.0012			ND	
OPEX total	2.48			1.71	
OPEX—PC	0.94			1.71	
Product capitalization (PC)	(NH_4_)_2_SO_4_ (30%) (*w*/*w*)			NA	
	1.54				

**Table 7:** Summary of the specifications for the membrane modules tested.

	Membrane 1	Membrane 2
Membrane material	PTFE	PP
Configuration (type)	Spiral wound	Spiral wound
Surface area (m^2^)	6.7	3.7
Flow feed side (m^3^/h)	0.05–0.7	0.05–1
Flow acid side (m^3^/h)	0.05–0.7	0.05–0.5
Pressure max (bar)	0.6	0.5
Temperature max (°C)	45	50

## Supplementary Materials

Supplementary materials can be found at https://www.mdpi.com/1422-0067/21/11/3914/s1.

## Abbreviations

NH_3_	Ammonia
WW	Wastewater
RCWW	Rendering condensate wastewater
NH_4_	Ammonium
PP	Polypropylene
PTFE	Polytetrafluorethylene
FOG	Fat, oil and grease
NaOH	Sodium hydroxide
((NH_4_)_2_SO_4_)	Ammonium sulphate
LEP	Liquid entry pressure
H_2_SO_4_	Sulfuric acid
CA	Contact angle
DI water	Deionised water
NaHCO_3_	Sodium bicarbonate
CaCO_3_	Calcium carbonate
CAPEX	Capital expenditure
OPEX	Operational expenditure
PC	Product capitalization
SEM	Scanning electron microscopy
AFM	Atomic force microscopy
