# Supplementary material for: Pilot Scale Study: First Demonstration of Hydrophobic Membranes for the Removal of Ammonia Molecules from Rendering Condensate Wastewater
Source: Int J Mol Sci. 2020 May 30;21(11):3914. doi: 10.3390/ijms21113914 (PMC7312626; doi:10.3390/ijms21113914)
Supplement: Supplementary file 1 [file ijms-21-03914-s001.pdf]

Supplementary information to support paper "Pilot scale study: First demonstration of hydrophobic membranes for the removal of ammonia from rendering condensate wastewater"

1. **Contact angle analysis on PTFE and PP membranes**

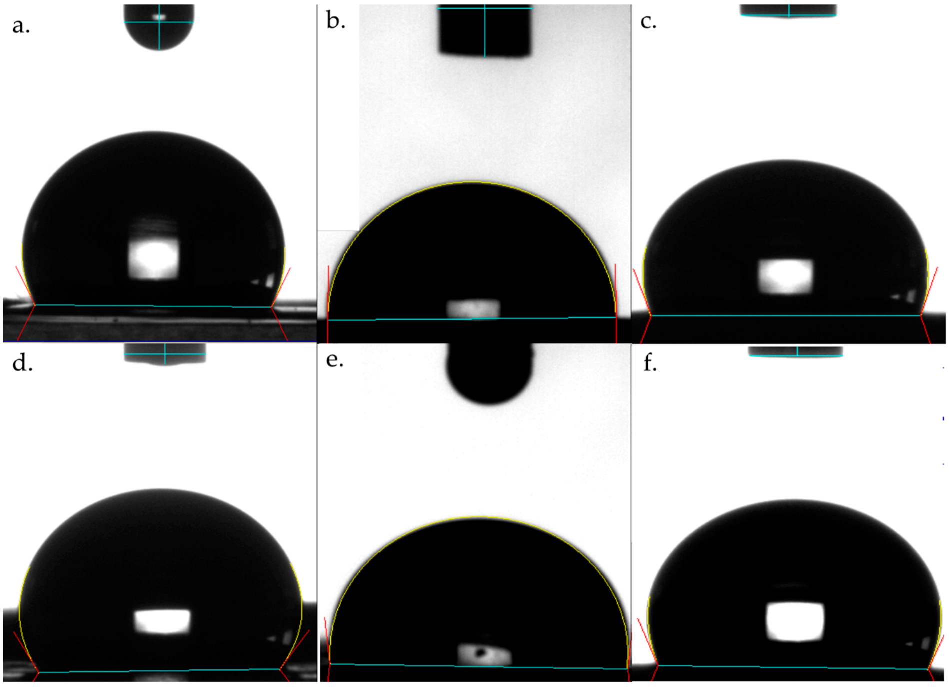

Figure 1: Droplets (1 μL) of DI water, RCWW and RCWW with FOG extracted on the PP (a-c) and PTFE (d-f) membrane showing the different surface wetting behaviour via the CA formed with the surface

Figure 1 consists of the DI water and RCWW droplet on the membrane in different states. It also contains an image of the needle at the top of each experiment which is used to calibrate the droplet to determine its contact angle with the surface.

2. AFM analysis of membrane surface roughness

Table 1: Surface roughness for PP samples at different exposure times to RCWW

Polypropylene				
Time (min)	RMS (nm)	Mean roughness (R_a)(nm)	R_{max} (nm)	Surface area (SA)(μm^2)
0	154.64	124.04	751	32.41
15	134.48	99.82	1716	38.54
30	132.86	112.64	640	32.57
45	511.33	442.71	2480	45.57
60	118.92	89.883	2231	43.13
120	962.2	832.74	3534	38.29
180	113.66	81.2	809	32.16
240	255.98	202.31	1217	34.61
300	109.49	90.37	753	28.39
360	86.71	66.07	759.76	24.05

Table 2: Surface roughness for PTFE samples at different exposure times to RCWW

Polytetrafluoroethylene				
Time (min)	RMS (nm)	Mean roughness (Ra)(nm)	Rmax (nm)	Surface area (SA)(μm^2)
0	249.23	203.92	1597	68.54
15	164.4	138.91	930	37.87
30	-	-	-	-
45	131.61	108.5	853	33.32
60	120.42	95.04	687	22.12
120	114.87	86.28	880	33.19
180	109.74	86.52	435	25.23
240	94.98	74.94	721	35.4
300	556.12	450.47	3252	58.55
360	85.57	65.48	584	32.89

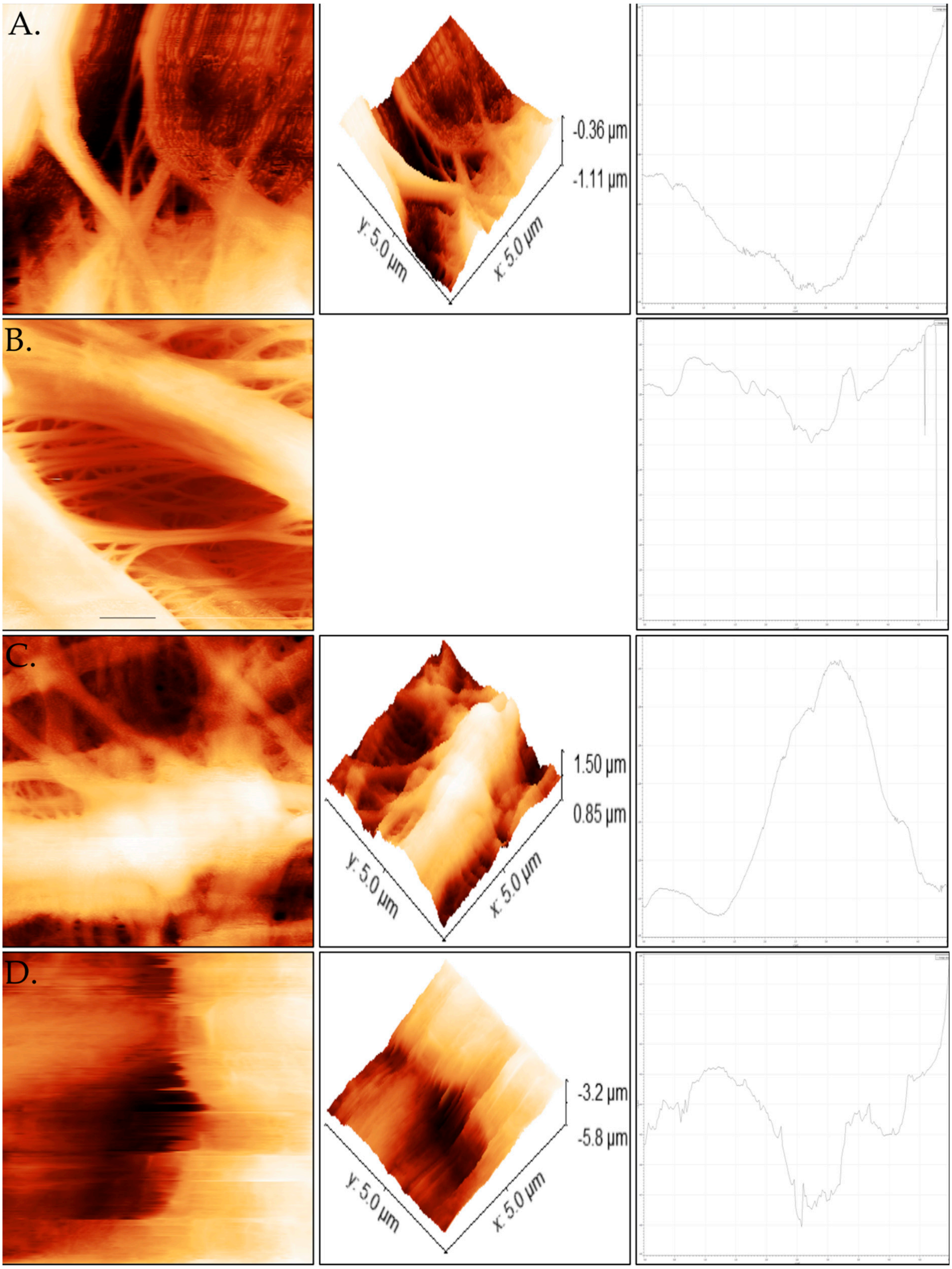

Figure 2: AFM analysis with flat maps, surface height maps and 2D profile, respectively for A) PP with no RCWW exposure, B) PP after 15 min RCWW exposure, C) PP after 30 min RCWW exposure and D) PP after 45 min exposure

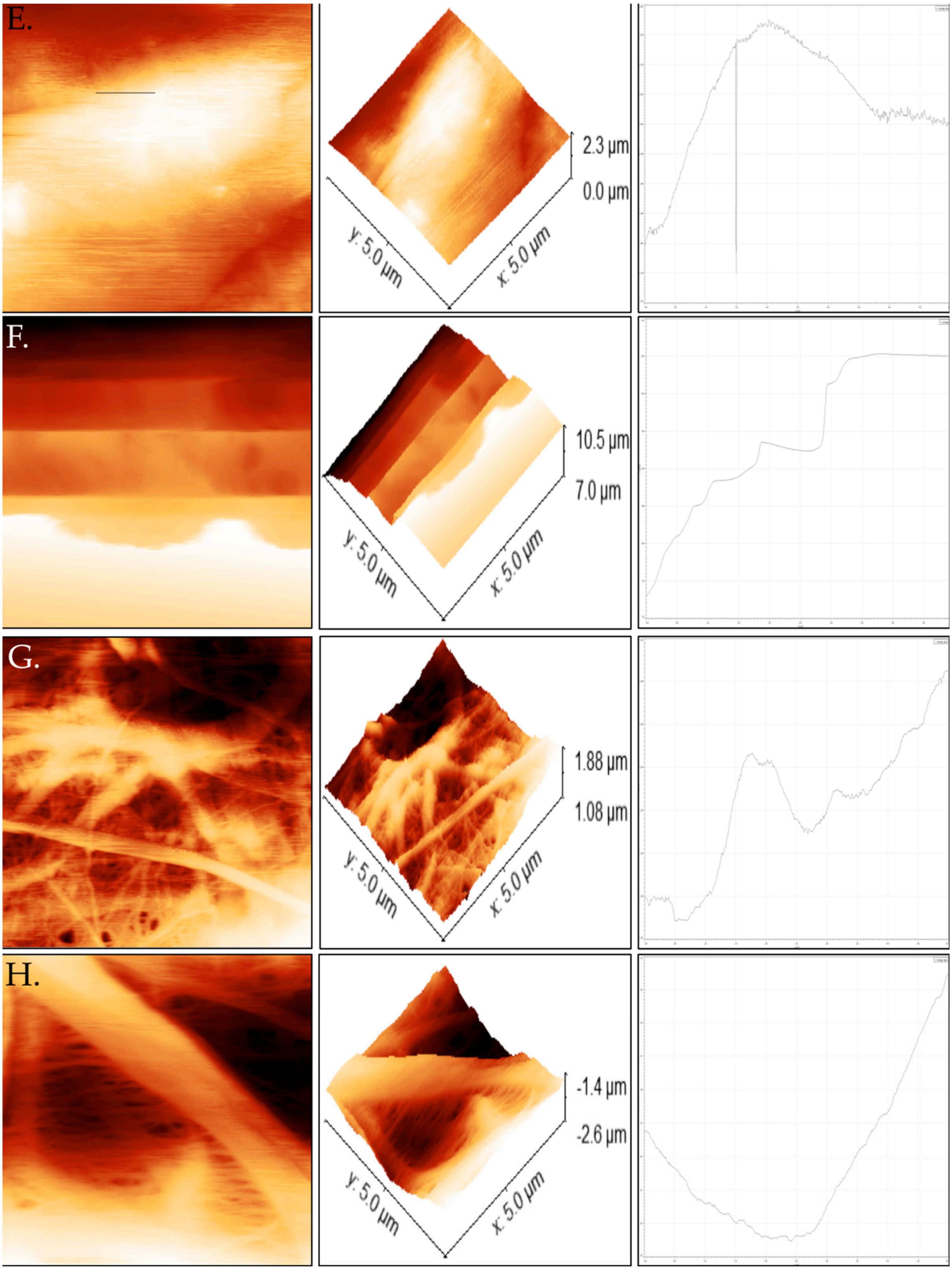

Figure 3: AFM analysis with flat maps, surface height maps and 2D profile, respectively for E) PP after 60 min RCWW exposure, F) PP after 120 min RCWW exposure, G) PP after 180 min RCWW exposure and H) PP after 240 min exposure

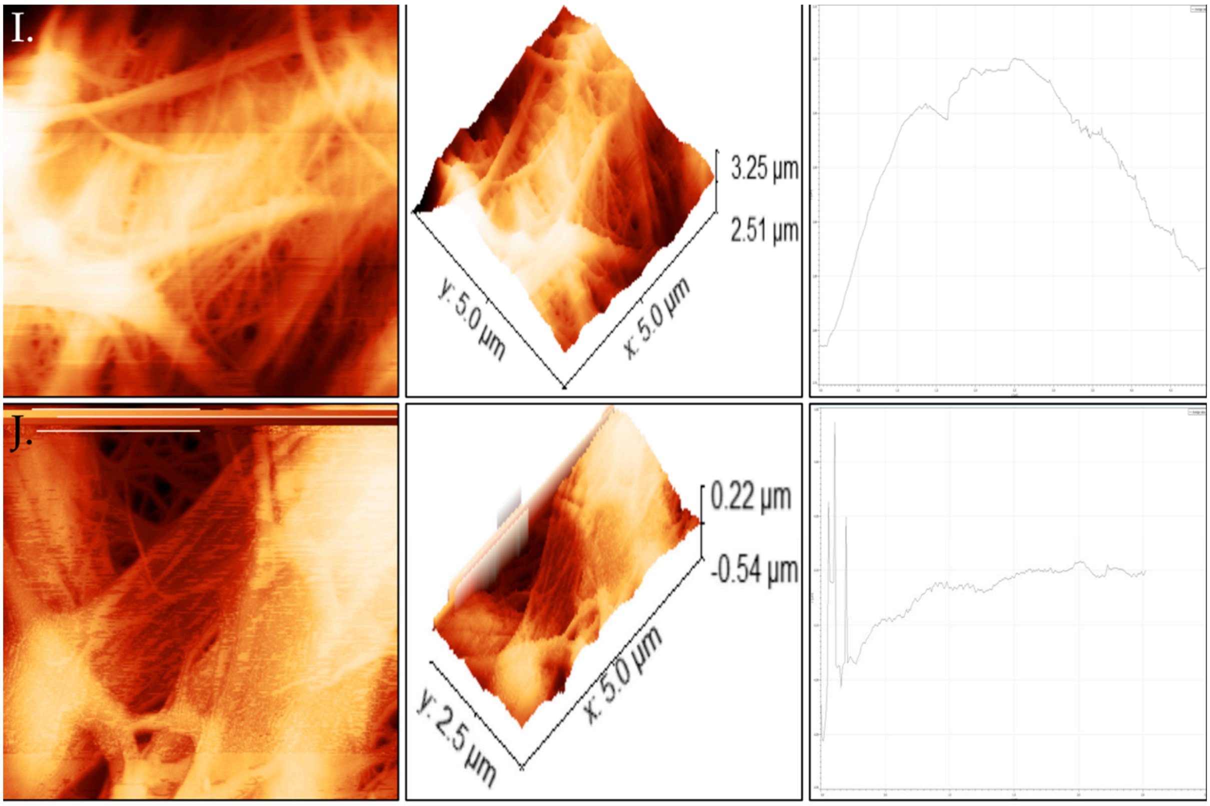

Figure 4: AFM analysis with flat maps, surface height maps and 2D profile, respectively for I) PP after 300 min RCWW exposure, J) PP after 360 min RCWW exposure

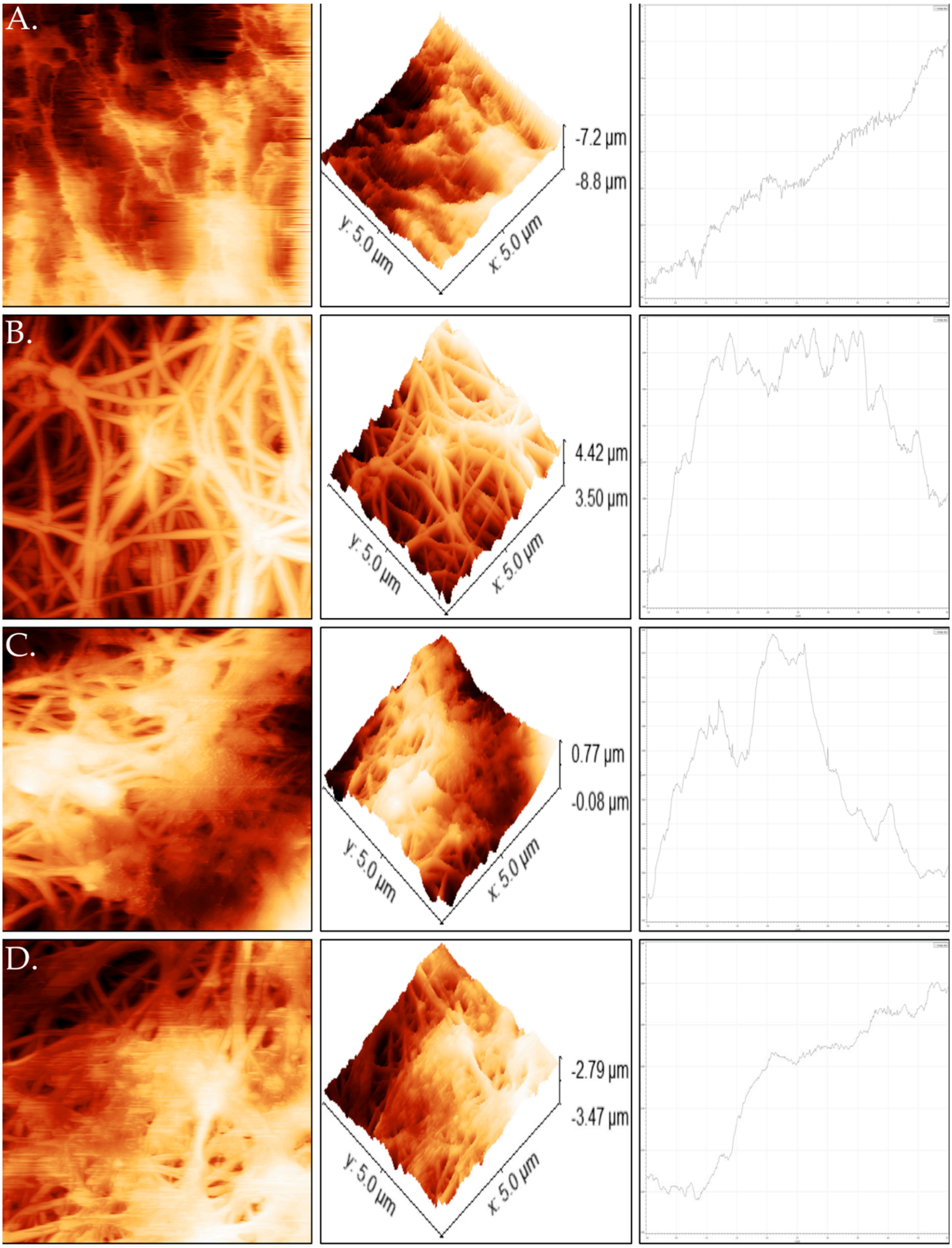

Figure 5: AFM analysis with flat maps, surface height maps and 2D profile, respectively for A) PTFE with no RCWW exposure, B) PTFE after 15 min RCWW exposure, C) PTFE after 45 min RCWW exposure and D) PTFE after 60 min exposure

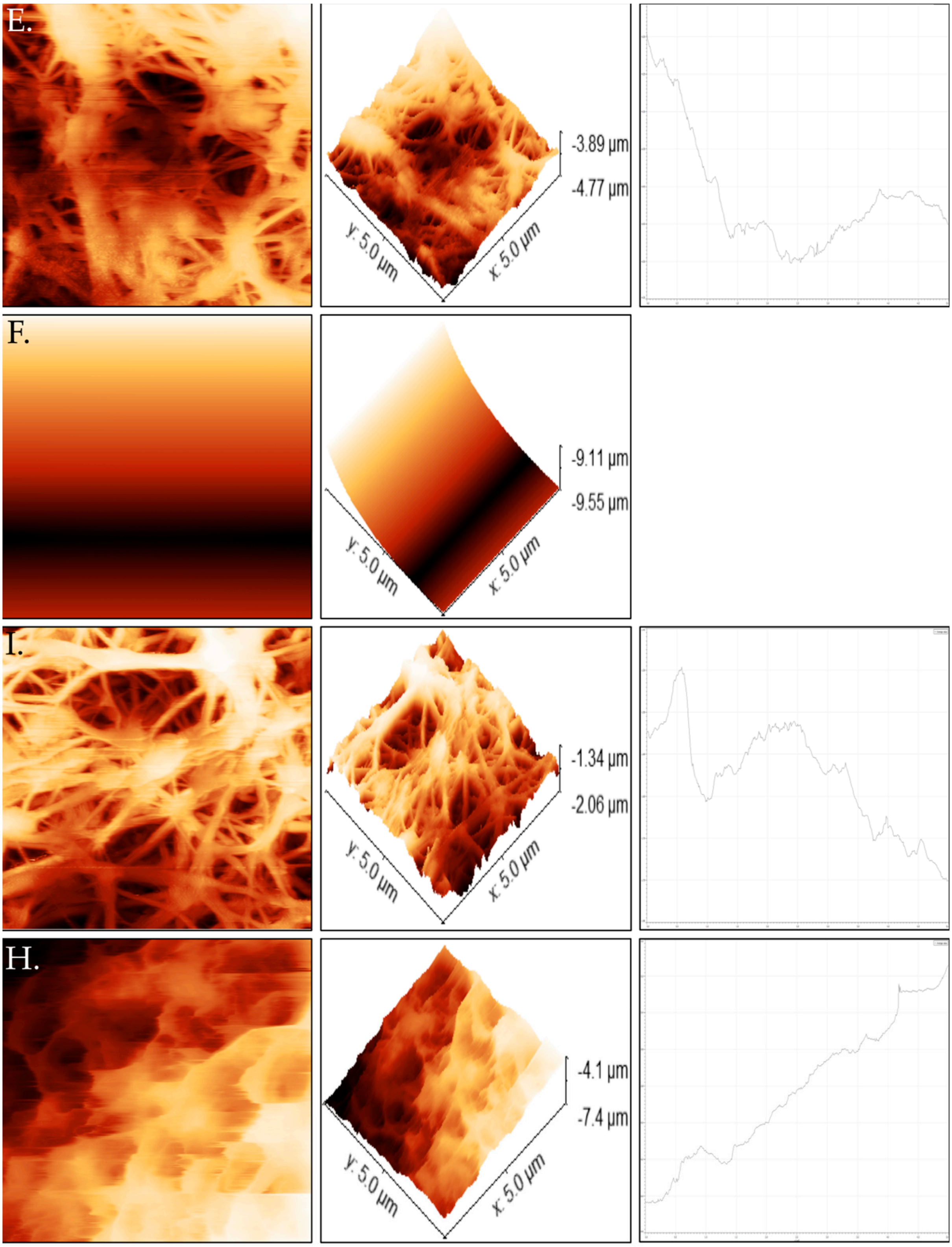

Figure 6: AFM analysis with flat maps, surface height maps and 2D profile, respectively for A) PTFE 120 min RCWW exposure, E) PTFE after 180 min RCWW exposure, F) PTFE after 240 min RCWW exposure and D) PTFE after 300 min exposure

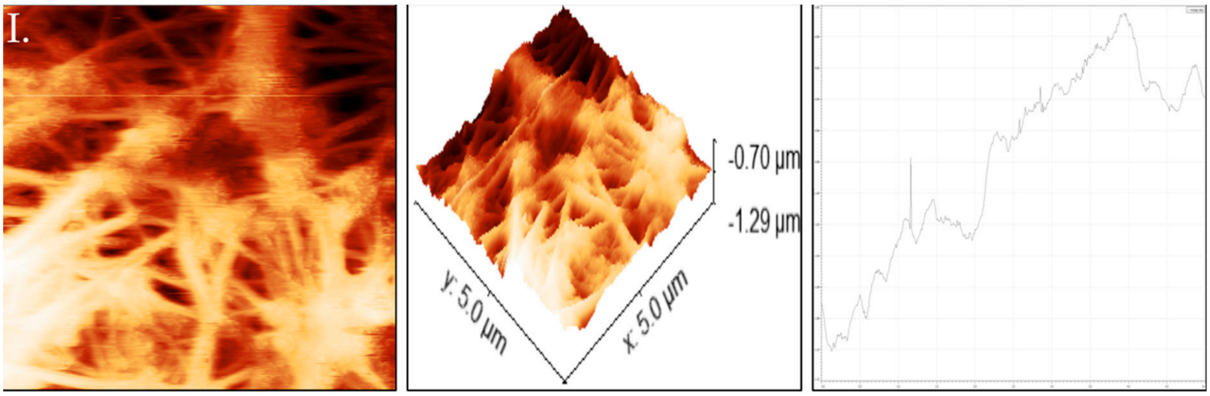

Figure 7: AFM analysis with flat maps, surface height maps and 2D profile, respectively for PTFE 360 min RCWW exposure
